# Heat shock protein 70 increases cell proliferation, neuroblast differentiation, and the phosphorylation of CREB in the hippocampus

**DOI:** 10.1186/s42826-019-0020-2

**Published:** 2019-11-01

**Authors:** Hyun Jung Kwon, Woosuk Kim, Hyo Young Jung, Min Soo Kang, Jong Whi Kim, Kyu Ri Hahn, Dae Young Yoo, Yeo Sung Yoon, In Koo Hwang, Dae Won Kim

**Affiliations:** 1 Department of Biochemistry and Molecular Biology, Research Institute of Oral Sciences, College of Dentistry, Gangneung-Wonju National University, Gangneung, 25457 South Korea; 2 Department of Biomedical Sciences, and Research Institute for Bioscience and Biotechnology, Hallym University, Chuncheon, 24252 South Korea; 3 Department of Anatomy and Cell Biology, College of Veterinary Medicine, and Research Institute for Veterinary Science, Seoul National University, Seoul, 08826 South Korea; 4 Department of Anatomy, College of Veterinary Medicine, Kangwon National University, Chuncheon, 24341 South Korea; 5 Department of Anatomy, College of Medicine, Soonchunhyang University, Cheonan, 31151 South Korea

**Keywords:** cAMP response element-binding protein, Cell proliferation, Heat shock protein 70, Hippocampus, Neuroblast differentiation, Novel object recognition

## Abstract

In the present study, we investigated the effects of heat shock protein 70 (HSP70) on novel object recognition, cell proliferation, and neuroblast differentiation in the hippocampus. To facilitate penetration into the blood–brain barrier and neuronal plasma membrane, we created a Tat-HSP70 fusion protein. Eight-week-old mice received intraperitoneal injections of vehicle (10% glycerol), control-HSP70, or Tat-HSP70 protein once a day for 21 days. To elucidate the delivery efficiency of HSP70 into the hippocampus, western blot analysis for polyhistidine was conducted. Polyhistidine protein levels were significantly increased in control-HSP70- and Tat-HSP70-treated groups compared to the control or vehicle-treated group. However, polyhistidine protein levels were significantly higher in the Tat-HSP70-treated group compared to that in the control-HSP70-treated group. In addition, immunohistochemical study for HSP70 showed direct evidences for induction of HSP70 immunoreactivity in the control-HSP70- and Tat-HSP70-treated groups. Administration of Tat-HSP70 increased the novel object recognition memory compared to untreated mice or mice treated with the vehicle. In addition, the administration of Tat-HSP70 significantly increased the populations of proliferating cells and differentiated neuroblasts in the dentate gyrus compared to those in the control or vehicle-treated group based on the Ki67 and doublecortin (DCX) immunostaining. Furthermore, the phosphorylation of cAMP response element-binding protein (pCREB) was significantly enhanced in the dentate gyrus of the Tat-HSP70-treated group compared to that in the control or vehicle-treated group. Western blot study also demonstrated the increases of DCX and pCREB protein levels in the Tat-HSP70-treated group compared to that in the control or vehicle-treated group. In contrast, administration of control-HSP70 moderately increased the novel object recognition memory, cell proliferation, and neuroblast differentiation in the dentate gyrus compared to that in the control or vehicle-treated group. These results suggest that Tat-HSP70 promoted hippocampal functions by increasing the pCREB in the hippocampus.

## Introduction

Neural stem cells (NSCs) are located in the subventricular zone of the lateral ventricle and in the subgranular zone of the dentate gyrus, and differentiate into new neurons in the olfactory bulb and dentate gyrus [1–3]. These cells are maintained throughout life, and neurogenesis in the dentate gyrus is very important because the hippocampus is the most susceptible region to neurological disorders including ischemia and Alzheimer’s diseases [4–7]. In the dentate gyrus, NSCs are located in the subgranular zone, differentiate into neuroblasts, and then migrate into the granule cell layer to become mature neurons [2]. Newly generated neurons help the hippocampus to adapt from new environmental needs and to respond to cognitive demands, which improves the acquisition of new skills, movement coordination, and emotional control [8, 9]. The number of NSCs decreased with age, stress, and chemical toxins, but can be increased by exercise, environmental enrichment, and neuropsychiatric drugs such as antidepressants [10–14]. The modification of NSC populations is critical for neural repair, and NSCs are believed to be a potential therapeutic approach for neurological disorders.

Expression of heat shock protein (HSP) is induced by abnormal conditions in various cell structures, and HSPs were named dependent on their molecular weight [15]. HSP70 is a molecular chaperone that is found in the cytosol, nucleus, endoplasmic reticulum, and mitochondria. HSP70 has diverse functions such as protein folding, protein translocation across membranes, and non-specific cytoprotection against various events [16–18]. In the nervous system, HSP70 and its modulators show neuroprotective effects against neurological disorders including Alzheimer’s disease, Parkinson’s disease, Huntington’s disease, and stroke [19–21]. Chronic administration of exogenous HSP70 improves learning and memory in old mice, and increases synaptophysin immunoreactivity in the brain compared to the control group [22]. HSP70 has a cAMP 26 responsive element (CRE) motif in its promoter region that can be activated by CRE binding protein (CREB) [23, 24], which is one of the most important pathways for long-term memory formation [25, 26]. However, there has been a lack of evidence on the effects of HSP70 on neurogenesis in the hippocampus.

The blood–brain barrier and plasma membrane are mostly impermeable to bioactive molecules such as peptides, proteins, and oligonucleotides because of their unique structures to protect the brain and cells from toxicants. Conversely, therapeutics with targets located within the blood-brain barrier and plasma membrane are usually ineffective [27]. The Tat peptide from the human immunodeficiency virus-1 (HIV-1) has a short, cationic, and arginine-rich sequence responsible for its translocation property [28]. Recently, HIV-1 and its Tat protein is thought to be one of main factors to impair cognitive function and adult hippocampal neurogenesis. Tat inhibits hippocampal neurogenesis in the mouse brain and significantly reduces the proliferation, migration, and differentiation of cultured neural precursor cells [29–31]. However, in a previous study, we demonstrated that a Tat-fusion protein was efficiently transduced into cells, and demonstrated functional activity across the blood–brain barrier in the spinal cord [32] and brain [33]. In addition, Tat-HSP70 showed neuroprotective effects against ischemic damage in mice [34] and inhibited inflammatory response induced by ischemia [35]. In the present study, we created a Tat-HSP70 fusion protein and observed the effects of Tat-HSP70 on novel object recognition memory as well as on cell proliferation and neuroblast differentiation in the hippocampal dentate gyrus.

## Material and method

### Experimental animals

Forty male C57BL/6 J mice (7 weeks of age) were purchased from Jackson Laboratory Co. Ltd. (Bar Harbor, ME, USA). Five animals were housed per cage in a conventional area under standard conditions at ambient temperature (22 ± 2 °C) and humidity (60 ± 5%) on a 12:12 h light/dark cycle with ad libitum access to food and water. Animal handling and care conformed to guidelines of current international laws and policies (National Institutes of Health Guide for the Care and Use of Laboratory Animals, Publication No. 85–23, 1985, revised 1996) and were approved by the Institutional Animal Care and Use Committee of Seoul National University (SNU-170807-10). All experiments were conducted with an effort to minimize the number of animals used and the physiological stress caused by the procedures employed. All experimental procedures were conducted according to ARRIVE guidelines [36].

### Construction of expression vectors

A cell-permeable Tat expression vector was prepared in the present laboratory as previously described [37]. The cDNA sequence for human HSP70 was amplified by polymerase chain reaction (PCR) using the following primer sequences: forward, 5′-CTC GAG ATG GCC AAA GCC-3′ and reverse, 5′-GGA TCC CTA ATC TAC CTC CTC-3′. PCR products were excised, eluted (Expin Gel; GeneAll Biotechnology Co., Ltd., Seoul, Korea), and ligated into a TA cloning vector (pGEM®-T easy vector; Promega Corporation, Madison, WI, USA) according to the manufacturer’s protocol. The purified TA vector containing human HSP70 cDNA was ligated into the Tat expression vector to produce a Tat-HSP70 fusion protein. In a similar fashion, a control HSP70 was constructed that expressed the HSP70 protein without Tat. To produce the Tat-HSP70 and control HSP70 proteins, the plasmid was transformed into *Escherichia coli* BL21 cells. The transformed bacterial cells were grown in 100 mL of lysogeny broth media at 37 °C to a D600 value of 0.5–1.0, and then induced with 0.5 mM isopropyl β-D-1-thiogalactopyranoside at 37 °C for 6 h. Harvested cells were lysed by sonication and purified using a Ni^2+^-nitrilotriacetic acid Sepharose affinity column (Qiagen, Inc.) and PD-10 column chromatography (GE Healthcare, Chicago, IL, USA). The purified protein concentrations were estimated using a Bradford assay [38].

Equal amounts of proteins were analyzed using 10% sodium dodecyl sulfate polyacrylamide gel electrophoresis. Analyzed proteins were electrotransferred to a polyvinylidene difluoride membrane, and then the membrane was blocked with tri-buffered saline (25 mM Tris-HCl, 140 mM NaCl, 0.1% Tween 20, pH 7.5) containing 5% non-fat dry milk. The membrane was probed using polyhistidine antibodies (1:2000, His-probe, SantaCruz Biotechnology, Santa Cruz, CA, USA). Proteins were identified using chemiluminescent reagents as recommended by the manufacturer (Amersham, Franklin Lakes, NJ, USA).

### Administration of tat-HSP70

The mice were divided into four groups: control, vehicle (10% glycerol)-treated, control-HSP70-treated, and Tat-HSP70-treated group. Vehicle, 5 nmol control-HSP70, or 5 nmol Tat-HSP70 was intraperitoneally administered to mice at 8 weeks of age, once a day for 3 weeks. This dosage was adapted because Tat-HSP70 in this dosage showed neuroprotective effects against Parkinson’s disease induced by 1-methyl-4-phenyl-1,2,3,6-tetrahydropyridine treatment in mice [39].

### Novel object recognition test

The testing apparatus consisted of an open box (25 cm × 25 cm × 25 cm) made of black acryl as described in the previous study [40]. The floor was covered with woodchip bedding, which was moved around between trials and testing days to prevent the build-up of odor in certain places. The objects to be discriminated were made of solid metal and could not be displaced by the mice due to their weight. The objects were cleaned with bleach to remove residual odors.

On the 20th day of treatment with vehicle, control-HSP70, or Tat-HSP70, at 1 h after treatment, mice from each group (*n* = 10 per group) were allowed to explore the apparatus for 2 min. On the testing day (21st day of treatment), two 2-min trials were performed 1 h following the last treatment. In the “sample” trial (T1), two identical objects were placed in two opposite corners of the apparatus. A mouse was placed in the apparatus and left to explore these two identical objects. After T1, the mouse was placed back in its home cage for an inter-trial interval of 1 h. Subsequently, a “choice” trial (T2) was performed. In T2, a new object replaced one of the objects that was present in T1. The mice were exposed again to two different (familiar and new) objects. Exploration was defined as directing the nose toward the object at a distance of no more than 2 cm and/or touching the object with the nose. From this measure, a series of variables were then calculated: the total time spent in exploring the two identical objects in T1, and the time spent in exploring two different objects in T2.

The distinction between familiar and new objects in T2 was determined by comparing the time spent exploring familiar object with that spent exploring new object. The discrimination index represents the difference in exploration time expressed as a proportion of the total time spent exploring the two objects in T2.

### Western blot

Following the novel object recognition test, animals in the control, vehicle-treated, control-HSP70-treated, and Tat-HSP70-treated groups (*n* = 5 in each group) were sacrificed and analyzed by western blotting as described previously [32, 40]. Following deep anesthesia with 2 g/kg urethane (Sigma-Aldrich, St. Louis, MO, USA) and subsequent decapitation, the hippocampal tissues were cut into 500-μm-thick sections on a vibratome (Leica Microsystems GmbH), and the hippocampus was cut out using a surgical blade. The hippocampal tissues were homogenized in 50 mM phosphate-buffered saline (PBS, pH 7.4) containing 0.1 mM ethylene glycol-bis(β-aminoethyl ether)-N,N,N′,N′-tetraacetic acid (pH 8.0), 0.2% Nonidet P-40, 10 mM ethylenediaminetetraacetic acid (pH 8.0), 15 mM sodium pyrophosphate, 100 mM β-glycerophosphate, 50 mM sodium fluoride, 150 mM sodium chloride, 2 mM sodium orthovanadate, 1 mM phenylmethylsulfonyl fluoride, and 1 mM dithiothreitol (DTT). Following centrifugation for 5 min at 16,000×*g* at 4 °C, the protein concentration was determined in the supernatants using a Micro BCA protein assay kit with bovine serum albumin as the standard (Pierce; Thermo Fisher Scientific, Inc., Waltham, MA, USA). Aliquots containing 20 μg of total protein were boiled in loading buffer containing 150 mM Tris (pH 6.8), 3 mM DTT, 6% sodium dodecyl sulfate, 0.3% bromophenol blue, and 30% glycerol. Each aliquot was subsequently loaded onto a polyacrylamide gel. Following electrophoresis, the proteins in the gel were transferred to a nitrocellulose membrane (Pall Life Sciences, Port Washington, NY, USA). To reduce background staining, the membrane was incubated with 5% non-fat dry milk in PBS containing 0.1% Tween-20 for 45 min at 25 °C, which was followed by incubation with rabbit anti-polyhistidine primary antibody (1:2000, His-probe, SantaCruz Biotechnology), rabbit anti-doublecortin (DCX) antibody (1:10,000; Abcam, Cambridge, UK), rabbit anti-phosphorylated CREB at Ser133 (pCREB; 1:1000; Cell Signaling Technology, Inc., Beverly, MA, USA), or rabbit anti-CREB (1:1000; Cell Signaling Technology, Inc.), peroxidase-conjugated goat anti-rabbit IgG (1:5000, SantaCruz Biotechnology), and an ECL chemiluminescent kit (Pierce; Thermo Fisher Scientific, Inc.).

### Tissue processing

Following the novel object recognition test, animals (*n* = 5 in each group) were deeply anesthetized with 1 g/kg of urethane (Sigma-Aldrich) and perfused transcardially with 0.1 M PBS (pH 7.4) followed by 4% paraformaldehyde in 0.1 M PBS (pH 7.4) as described previously [32, 40]. The brains were dissected and post-fixed for 12 h in the same fixative. The tissue was cryoprotected by overnight saturation with 30% sucrose. Serial brain sections were cut coronally at a thickness of 30 μm using a cryostat (Leica, Wetzlar, Germany), and collected in 6-well plates containing PBS for further processing.

### Immunohistochemistry

All sections were processed under the same conditions to ensure that the immunohistochemical data were comparable among the groups. Tissue sections located at a distance of 90 μm from each other were selected from an area between 1.82 and 2.30 mm posterior to the bregma, as defined by a mouse atlas [41] for Ki67, DCX, and pCREB immunohistochemistry. The sections were sequentially treated with 0.3% H_2_O_2_ in PBS for 30 min and 10% normal goat serum in 0.05 M PBS for 30 min at 25 °C. Sections first underwent an overnight incubation with mouse anti-HSP70 antibody (1:500; Calbiochem, EMD Millipore, Temecula, CA, USA), rabbit anti-Ki67 antibody (1:1000; Abcam), rabbit anti-DCX (1:5000; Abcam), or rabbit anti-pCREB (1:400; Cell Signaling Technology, Inc.) at 25 °C. Thereafter, the sections were treated with biotinylated goat anti-mouse or anti-rabbit IgG and a streptavidin-peroxidase complex (1:200; Vector, Burlingame, CA, USA) for 2 h at 25 °C. Sections were visualized by reaction with 3,3′-diaminobenzidine tetrachloride (Sigma) in 0.1 M Tris-HCl buffer (pH 7.2) and mounted on gelatin-coated slides. Sections were dehydrated and mounted with Canada balsam (Kanto Chemical, Tokyo, Japan).

### Data analysis

Analysis of the hippocampal dentate gyrus for DCX was performed using an image analysis system and ImageJ software v. 1.50 (National Institutes of Health, Bethesda, MD, USA). Data analysis was carried out under the same conditions by two observers for each experiment to ensure objectivity in blinded conditions as described in the previous study [40]. Tissue sections located at a distance of 90 μm from each other were selected from an area between 1.82 and 2.30 mm posterior to the bregma, as defined by a mouse atlas [41]. Digital images of the whole dentate gyrus were captured with a BX51 light microscope (Olympus, Tokyo, Japan) equipped with a digital camera (DP72, Olympus) connected to a computer monitor. Images were calibrated into an array of 512 × 512 pixels corresponding to a tissue area of 1200 μm × 900 μm (100× primary magnification). Each pixel resolution had 256 Gy levels, and the intensity of DCX immunoreactivity was evaluated by relative optical density (ROD), which was obtained after transformation of the mean gray level using the following formula: ROD = log (256/mean gray level). The ROD of background staining was determined in unlabeled portions of the sections using Photoshop CC 2018 software (Adobe Systems Inc., San Jose, CA, USA), and this value was subtracted to correct for nonspecific staining using ImageJ v. 1.50 software (National Institutes of Health). Data are expressed as a percentage of the control group (which was set at 100%).

The Ki67- and pCREB-immunoreactive nuclei in the whole dentate gyrus was counted using an analysis system equipped with a computer-based CCD camera (OPTIMAS software version 6.5; CyberMetrics® Corporation, Phoenix, AZ, USA; magnification, 100×) as described in a previous study [42]. The image was converted to a gray-scale image, and Ki67- and pCREB-immunoreactive nuclei were automatically selected according to the intensity of the immunohistochemical staining for Ki67 and pCREB, respectively.

### Statistical analysis

The data were expressed as the mean of the experiments performed for each experimental investigation. In order to determine the changes of cell number and ROD, mean differences among the groups were analyzed statistically by one-way analyses of variance followed by Bonferroni’s post-hoc test using GraphPad Prism 5.01 software (GraphPad Software, Inc., La Jolla, CA, USA). The results were considered to be statistically significant if *p* < 0.05.

## Results

### Construction of control-HSP and tat-HSP70 fusion proteins

**Fig. 1:**
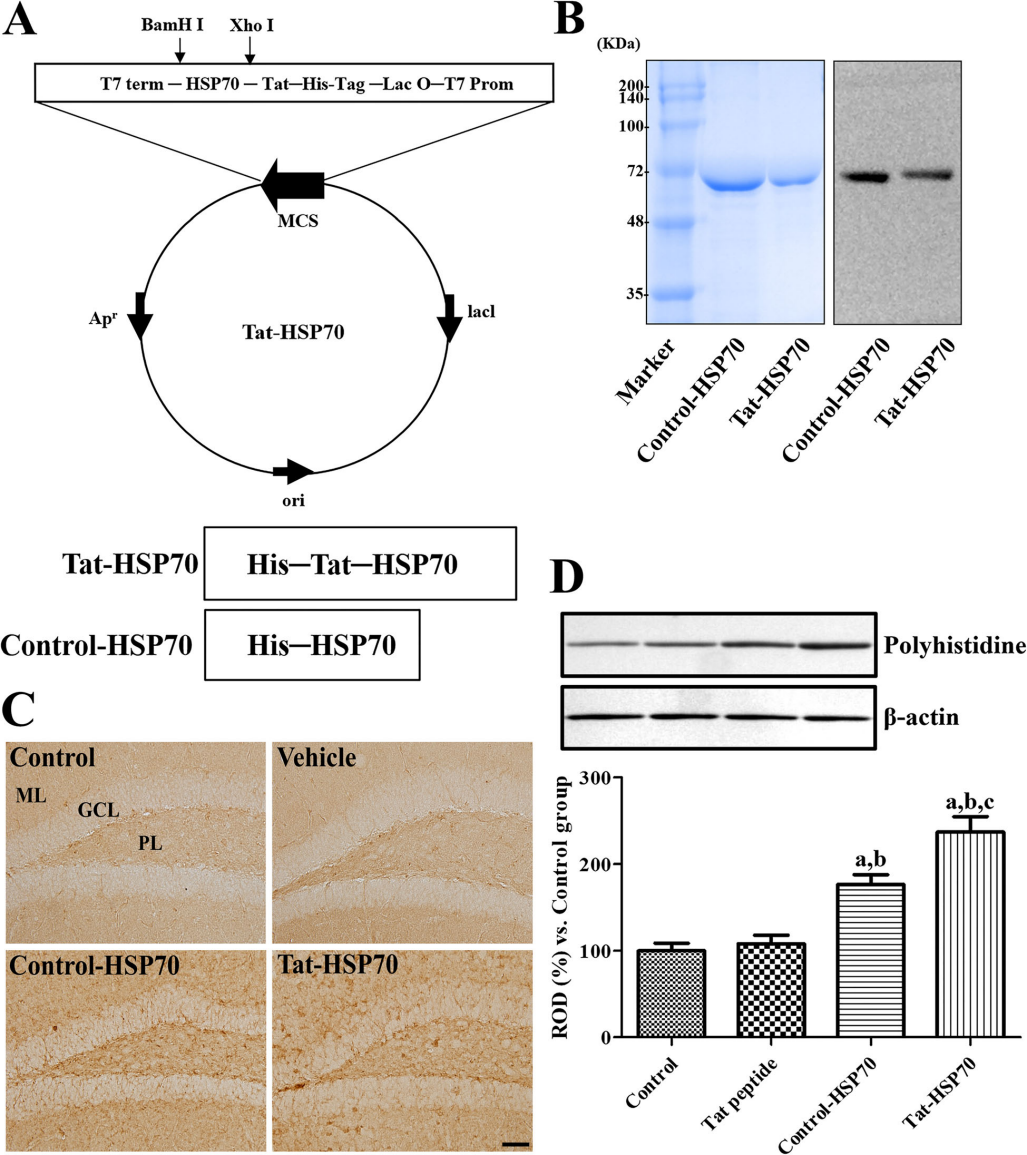
Purification and transduction of the Tat-HSP70 protein into the mouse hippocampus. **a** Overview of the Tat-HSP70 protein. **b** Expression and purification of the Tat-HSP70 and control-HSP70 proteins, as assessed by western blot analyses using an anti-polyhistidine antibody. **c** Western blot analysis of polyhistidine in the hippocampal homogenates of control, vehicle (10% glycerol)-, control-HSP70-, and Tat-HSP70-treated groups (*n* = 5 per group; ^a^*P* < 0.05, versus control group; ^b^*P* < 0.05, versus vehicle-treated group; ^c^*P* < 0.05, versus control-HSP70 treated group). **d** Immunohistochemistry for HSP70 in the dentate gyrus of control, vehicle-, control-HSP70-, and Tat-HSP70-treated mice. Note that HSP70 immunoreactive structures are most abundantly found in the Tat-HSP70-treated group. GCL, granule cell layer; ML, molecular layer; PL, polymorphic layer. Scale bar = 50 μm

To produce the control-HSP70 and Tat-HSP70 fusion proteins, human HSP70 genes were fused to a Tat peptide expression vector, and the control-HSP70 protein was manufactured without a Tat domain (Fig. 1a). After the overexpression of vectors, purified control-HSP70 and Tat-HSP70 fusion proteins were obtained by Ni^b+^- → Ni^2+^- nitrilotriacetic acid Sepharose affinity column and PD-10 column chromatography. Western blot analysis revealed strong bands for control-HSP and Tat-HSP70 (Fig. 1b).

### Confirmation of tat-HSP70 delivery into the hippocampus

The delivery of Tat-HSP70 was assessed by western blot analysis for polyhistidine and by immunohistochemistry for HSP70. Polyhistidine protein levels were significantly increased in the hippocampal homogenates of control-HSP70 and Tat-HSP70-treated group compared to that in control or vehicle-treated group. In addition, polyhistidine protein levels were significantly higher in the Tat-HSP70-treated group compared to that in the control-HSP70-treated group (Fig. 1c). HSP70 immunoreactivity was faintly observed in the dentate gyrus of control and vehicle-treated group, while HSP70 immunoreactivity was abundantly observed in the dentate gyrus of control-HSP70 or Tat-HSP70-treated group. In addition, HSP70 immunoreactive structures were more abundant in the dentate gyrus of Tat-HSP70-treated group compared to that in the control-HSP70-treated group (Fig. 1d).

### Effects of tat-HSP70 on novel object recognition memory

**Fig. 2:**
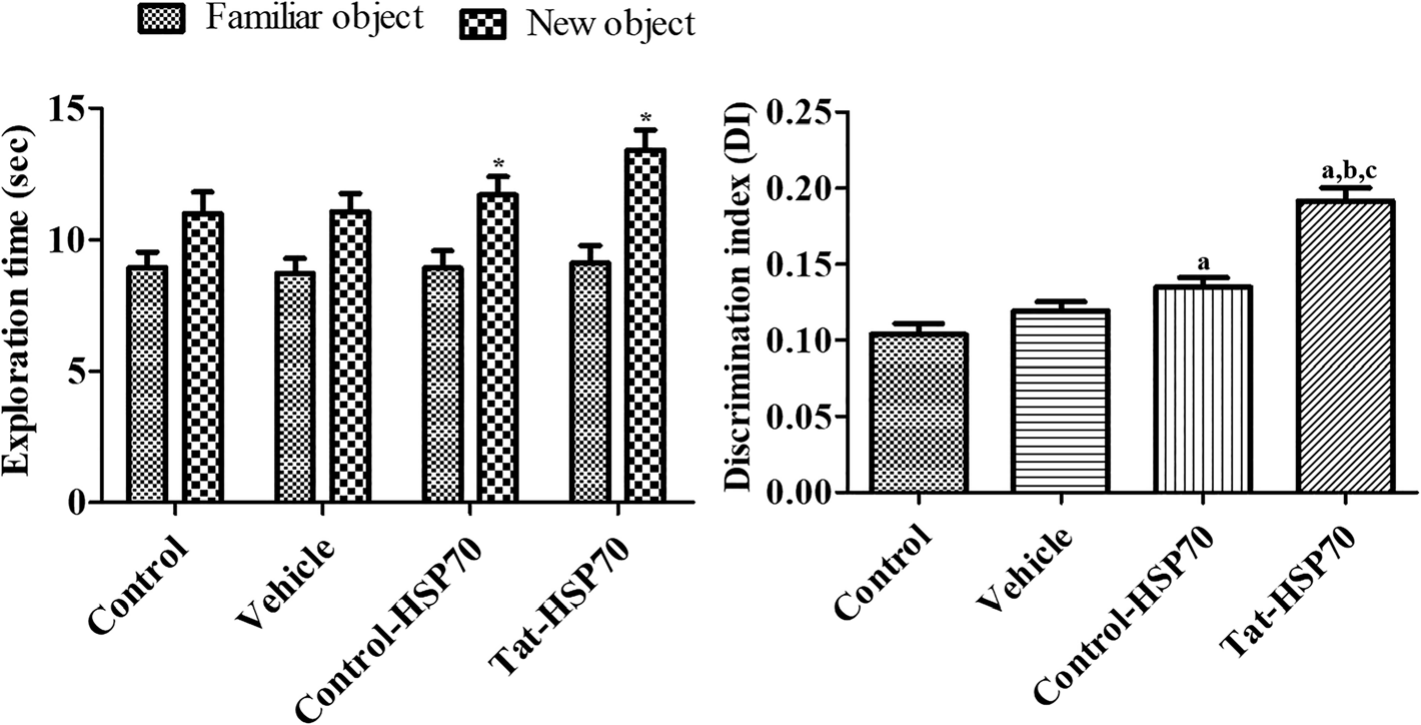
Exploration time (*n* = 10 per group; *p < 0.05, significant difference between familiar and novel objects) and discrimination index (*n* = 10 per group; ^a^*P* < 0.05, versus control group; ^b^*P* < 0.05, versus vehicle-treated group; ^c^*P* < 0.05, versus control-HSP70 treated group) of familiar vs. novel objects during a novel object recognition test in control, vehicle-, control-HSP70-, and Tat-HSP70-treated mice. Data of the exploration time for each object (same object, where one object was replaced by a new one on the testing day) are presented as a percentage of total exploration time. All data are shown as % exploration time ± SEM

During the training period, there were no significant differences on the amount of time spent exploring two identical objects among the groups. During the testing period, mice in all groups spent more time exploring the new object compared with a familiar one. However, mice in the control-HSP70- and Tat-HSP70-treated groups spent significantly more time exploring the new object compared to that in the control or vehicle-treated groups. The discrimination index was also significantly higher in the control-HSP70- and Tat-HSP70-treated groups compared to that in the control or vehicle-treated groups. The discrimination index was significantly higher in the Tat-HSP70-treated group compared to that in the control-HSP70-treated group (Fig. 2).

### Effects of tat-HSP70 on cell proliferation in the dentate gyrus

**Fig. 3:**
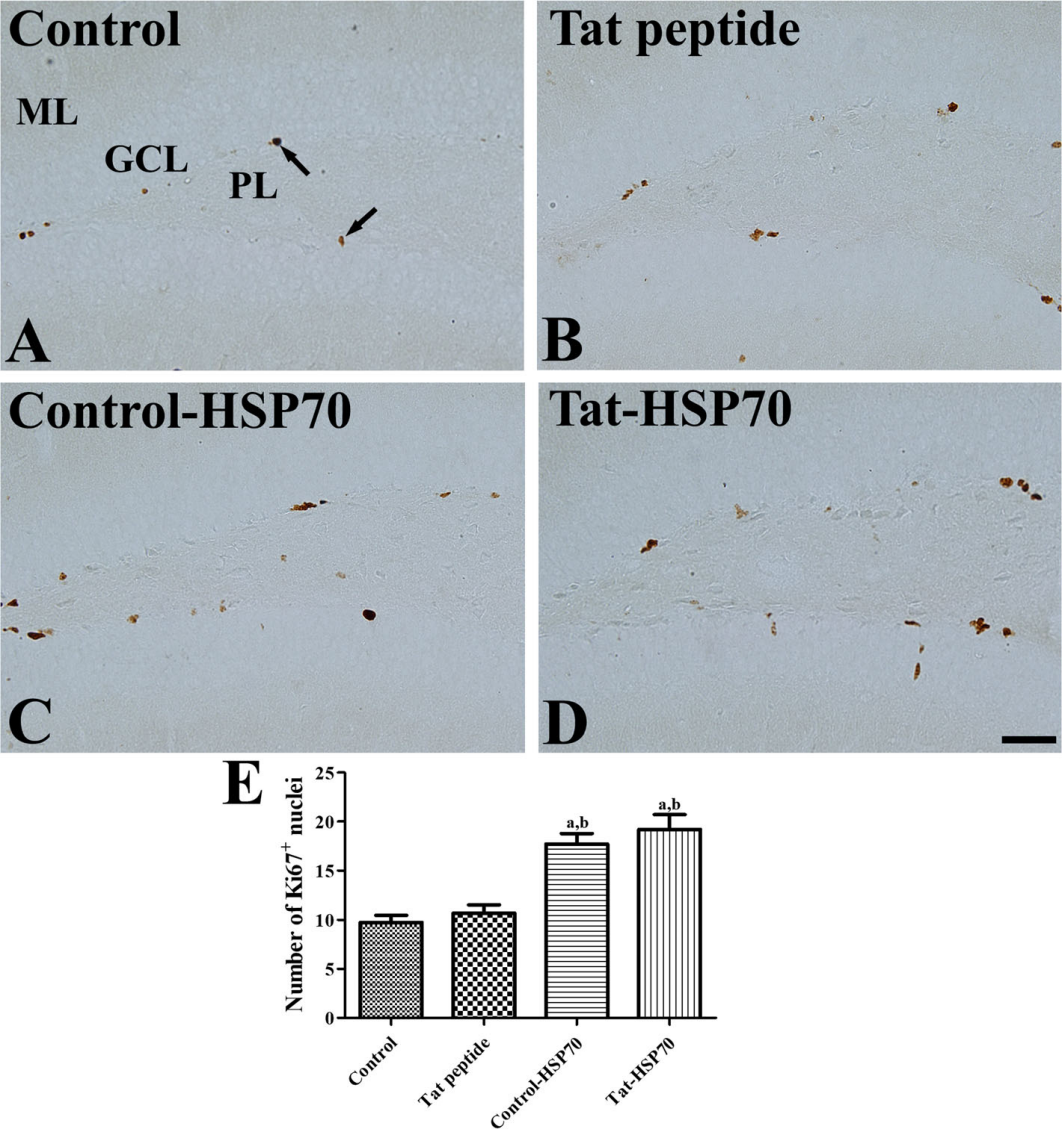
Immunohistochemistry for Ki67 in the dentate gyrus of control (**a**), vehicle- (**b**), control-HSP70- (**c**), and Tat-HSP70-treated (**d**) mice. In all groups, Ki67-positive nuclei (arrows) are found in the subgranular zone of the dentate gyrus. Note that Ki67-positive nuclei are abundantly detected in the dentate gyrus of control-HSP70 and Tat-HSP70-treated mice. GCL, granule cell layer; ML, molecular layer; PL, polymorphic layer. Scale bar = 50 μm. **e** The number of Ki67-positive nuclei in the dentate gyrus per section for each group are also shown (*n* = 5 per group; ^a^*P* < 0.05, versus control group; ^b^*P* < 0.05, versus vehicle-treated group). Data are presented as mean ± SEM

In all groups, Ki67-positive nuclei were mainly found in the subgranular zone of dentate gyrus, but the number of Ki67-positive nuclei was significantly different among groups. In the control and vehicle-treated groups, some Ki67 nuclei was observed in the dentate gyrus (Fig. 3a and b), and the number of Ki67-positive nuclei was slightly increased in the vehicle-treated group compared to that in the control group, although the statistical significance was not detected between the control and vehicle-treated group (Fig. 3e). In the control-HSP70- and Tat-HSP70-treated groups, Ki67-positive nuclei were abundant compared to that in the control group (Fig. 3c and d). The number of Ki67-positive nuclei in the control-HSP70- and Tat-HSP70-treated groups was significantly increased by 181.9 and 197.1% of the control group, respectively (Fig. 3e).

### Effects of tat-HSP70 on neuroblast differentiation in the dentate gyrus

**Fig. 4:**
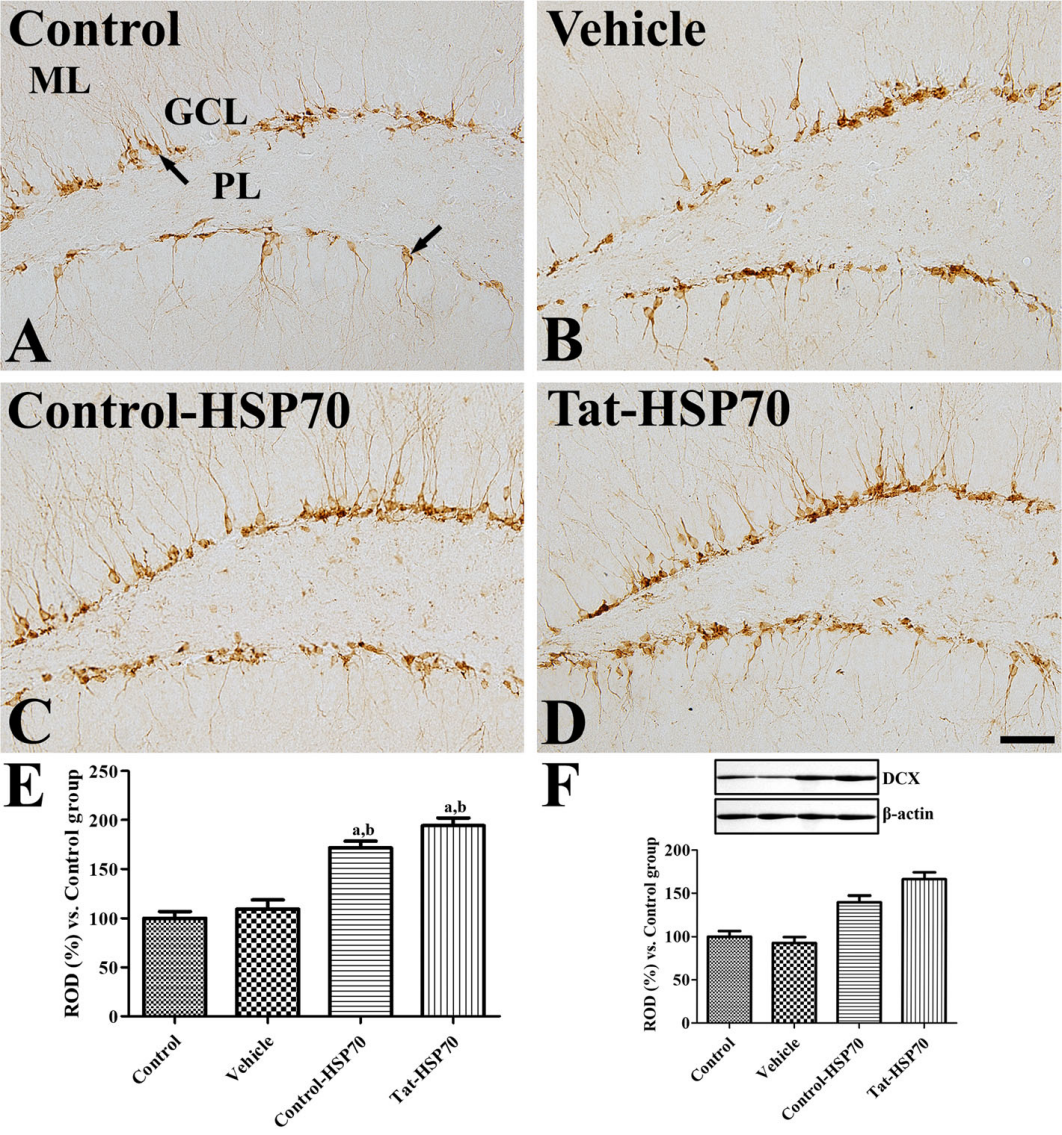
Immunohistochemistry for DCX in the dentate gyrus of control (**a**), vehicle- (**b**), control-HSP70- (**c**), and Tat-HSP70-treated (**d**) mice. In all groups, DCX-immunoreactive neuroblasts (arrows) are found in the dentate gyrus with dendrites. Note that DCX-immunoreactive neuroblasts shows delicate dendritic tree branching in the dentate gyrus of control-HSP70- and Tat-HSP70-treated mice. GCL, granule cell layer; ML, molecular layer; PL, polymorphic layer. Scale bar = 50 μm. **e** The relative optical densities (RODs) expressed as a percentage of the value representing the DCX immunoreactivity in the dentate gyrus of the control group are shown. **f** Values from western blot analysis is expressed as a ratio of DCX and β-actin immunoblot band in control group (*n* = 5 per group; ^a^*P* < 0.05, versus control group; ^b^*P* < 0.05, versus vehicle-treated group). Data are presented as mean ± SEM

In all groups, DCX immunoreactive neuroblasts were found in the dentate gyrus. Their cell bodies were located in the subgranular zone of dentate gyrus, and their dendrites extended into the molecular layer of dentate gyrus. However, DCX immunoreactivity was significantly different among the groups. In the control and vehicle-treated groups, DCX-immunoreactive neuroblasts were found sparsely in the dentate gyrus (Fig. 4a and b), and the DCX immunoreactivities were similar in the dentate gyrus between the groups (Fig. 4e). In the control-HSP70- and Tat-HSP70-treated groups, DCX-immunoreactive neuroblasts were abundantly found in the dentate gyrus, and DCX immunoreactivities in the control-HSP70- and Tat-HSP70-treated groups were significantly increased by 171.5 and 194.2% of the control group, respectively (Fig. 4c, d, and e). DCX protein levels showed significant increases in the control-HSP70 and Tat-HSP70-treated group compared to that in the control group and were highest in the Tat-HSP70-treated group (Fig. 4f).

### Effects of tat-HSP70 on the phosphorylation of CREB in the dentate gyrus

**Fig. 5:**
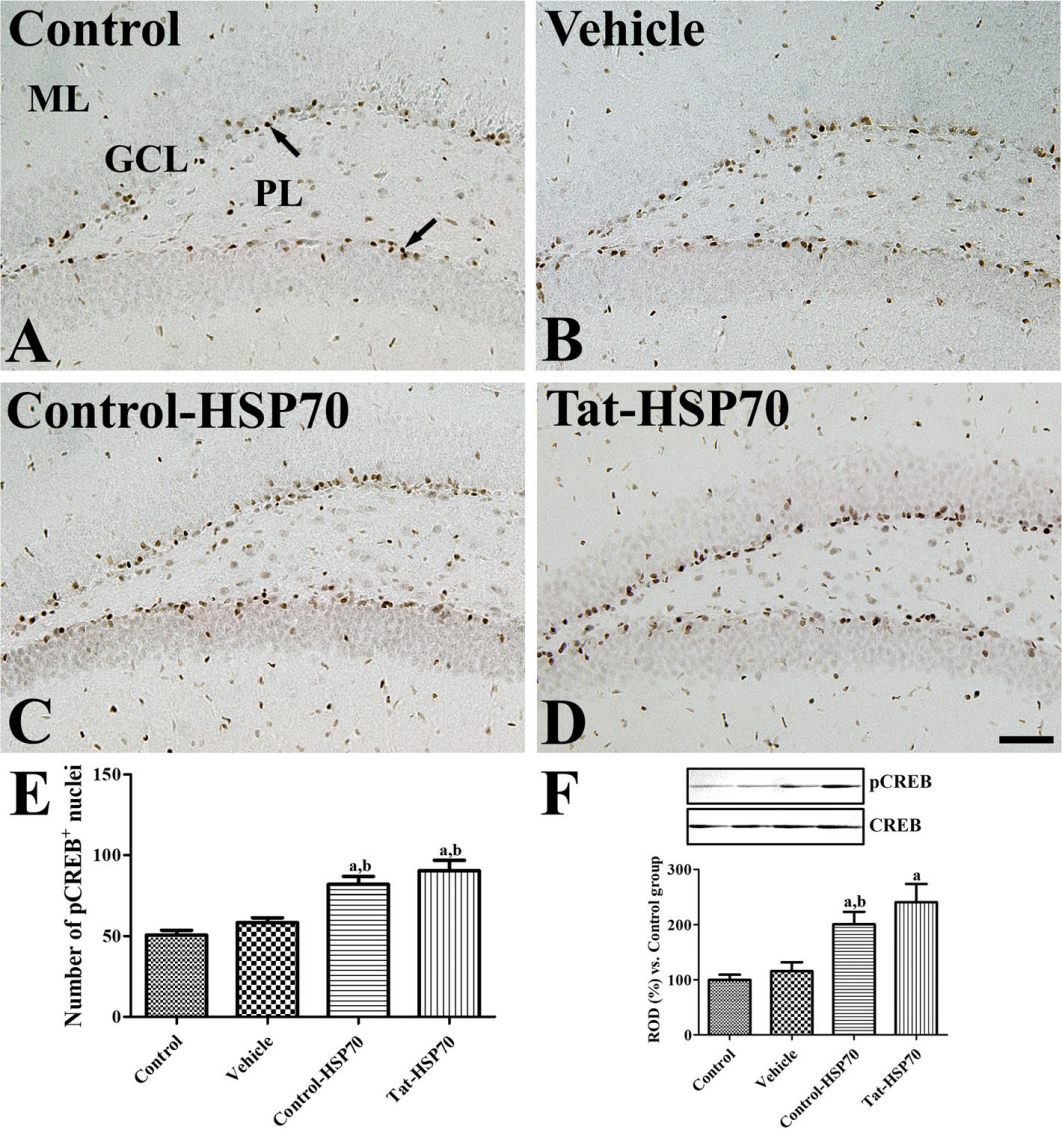
Immunohistochemistry for pCREB in the dentate gyrus of control (**a**), vehicle- (**b**), control-HSP70- (**c**), and Tat-HSP70-treated (**d**) mice. In all groups, pCREB-positive nuclei (arrows) are mainly observed in the subgranular zone of the dentate gyrus. Note that pCREB-positive nuclei are strongly observed in the dentate gyrus of Tat-HSP70-treated mice. GCL, granule cell layer; ML, molecular layer; PL, polymorphic layer. Scale bar = 50 μm. **e** The number of pCREB-positive nuclei per section for each group are shown and **f** values from western blot analysis is expressed as a ratio of pCREB and CREB immunoblot band in control group (*n* = 5 per group; ^a^*P* < 0.05, versus control group; ^b^*P* < 0.05, versus vehicle-treated group). Data are presented as mean ± SEMs

In all groups, pCREB-immunoreactive nuclei were mainly found in the subgranular zone of the dentate gyrus. However, the number of pCREB-immunoreactive nuclei was significantly different among the groups. A similar pattern of distribution and the number of pCREB-immunoreactive nuclei was found in the control and vehicle-treated groups (Fig. 5a, b, and e). In the control-HSP-treated group, pCREB-immunoreactive nuclei were abundant compared to that of the control group, although the statistical significance was not detected between the control and control-HSP70-treated groups (Fig. 5c and e). In the Tat-HSP70-treated group, pCREB-immunoreactive nuclei were strongly observed in the subgranular zone of the dentate gyrus, and the number of pCREB-immunoreactive nuclei was significantly increased in the dentate gyrus by 161.5 and 178.3% of the control group, respectively (Fig. 5d and e). Western blot study showed the significant increases in pCREB/CREB ratio in the control-HSP70 or Tat-HSP70-treated groups compared to that in the control or vehicle-treated group. However, there were no significant differences on the ratio of pCREB/CREB between control-HSP70 or Tat-HSP70-treated groups (Fig. 5f).

## Discussion

*hsp70* can regulate expression via negative feedback [43, 44], and overexpression of *HSPA1A* suppresses HSP70 induction. Several lines of evidence demonstrate that exogenous HSP70 can cross the blood–brain barrier and protects neurons from damage due to energy deprivation [45], Alzheimer’s disease [46], and epilepsy [47]. However, these studies have been conducted in pathological non-physiological conditions. In the present study, we made a Tat-HSP70 fusion protein and control-HSP70 protein to deliver HSP70 into neurons, and compared the effects of control-HSP70 and Tat-HSP70 on novel object recognition memory as well as cell proliferation and neuroblast differentiation in the dentate gyrus.

We successfully constructed control-HSP70 and Tat-HSP70 fusion proteins as indicated by the western blot analysis for polyhistidine, which showed differences in expression of polyhistidine at 1.56-kDa according to the molecular weight of the Tat peptide. To assess the delivery efficiency of HSP70 into the hippocampus, we administered control-HSP70 and Tat-HSP70 to mice for 21 days and observed significant increases in the expression of polyhistidine in both groups compared to that in the control or vehicle-treated group. However, polyhistidine protein levels were more abundantly found in the hippocampal homogenates of the Tat-HSP70-treated group compared to that in the control-HSP70-treated group. In addition, we also conducted the immunohistochemical staining for HSP70 to elucidate direct transfer of HSP70 in the hippocampus. Treatment with control-HSP70 or Tat-HSP70 showed significant increases in HSP70-immunoreactive structures compared to that in the control or vehicle-treated group although higher protein levels of HSP70 were observed in the Tat-HSP70-treated group than in the control-HSP70-treated group. This result suggests that HSP70 protein can cross the blood–brain barrier in naïve mice, and that Tat-HSP70 was efficiently delivered to the mouse hippocampus.

In the present study, administration of control-HSP70 or Tat-HSP70 for 3 weeks significantly enhanced the novel object recognition in the mice. This result was consistent with previous studies showing that exogenous HSP70 or HSP70 induces improvements in learning and memory formation [22, 48, 49]. Intranasal administration of exogenous HSP70 for 5 and 9 months resulted in improvements in learning and memory in old mice [22]. In addition, infusion of recombinant HSP70 (hspa1a) into the dorsal hippocampus facilitates memory consolidation immediately after training, while administration of HSP70 antibody blocked the memory consolidation immediately after training [49]. Geranylgeranylacetone, an inducer of HSP70, enhanced recovery of cognitive/affective function in the Morris water maze and novel object recognition in a mouse model of traumatic brain injury [48]. HSP70 has been found on the external leaflet of neuroblast membranes and regulate neuroblast migration in the subventricular zone [50]. Administration of control-HSP70 or Tat-HSP70 for 3 weeks significantly increased cell proliferation and neuroblast differentiation in the dentate gyrus. However, administration of geranylgeranylacetone for 8 weeks did not show any significant changes in DCX-immunoreactive neuroblasts in non-stress (control) mice, although they significantly increased DCX-immunoreactive neuroblasts in the stressed mice [51]. In the ischemic animals, Tat-HSP70-transduced neural precursor cells enhanced the functional outcome by preventing secondary neuronal degeneration as well as facilitating neurogenesis with sustained high levels of growth factors including brain-derived neurotrophic factor [52]. In an animal model for Alzheimer’s disease, intranasal administration of exogenous HSP70 increases genes involved in neuronal activity and neurogenesis [53].

To elucidate the possible mechanisms of HSP70 for facilitating cell proliferation and neuroblast differentiation in the dentate gyrus, we have focused on the CREB pathway because activation of CREB is one of the most important pathways for long-term memory formation and memory consolidation [25, 26]. Administration of control-HSP70 or Tat-HSP70 for 3 weeks significantly increased the number of pCREB-immunoreactive nuclei in the subgranular zone of dentate gyrus. This result suggests that control-HSP70 and Tat-HSP70 facilitated cell proliferation and neuroblast differentiation in the dentate gyrus by facilitating the phosphorylation of CREB. CREB is an essential factor for inducing the formation of synapses, long-term potentiation, and memory formation in mice [54–56]. In addition, we also observed that pCREB expression in the subgranular zone of dentate gyrus was mainly found in the DCX-immunoreactive neuroblasts in mice [57]. As CREB is a downstream regulator of the extracellular signal-regulated kinases (ERK) cascade, direct infusion of recombinant HSP70 into the dorsal hippocampus enhanced ERK activity immediately after training in the hippocampus of aged mice [49].

## Conclusion

Administration of Tat-HSP70 significantly improved novel object recognition by increasing cell proliferation and neuroblast differentiation in the dentate gyrus via the enhanced phosphorylation of CREB.

## Data Availability

The datasets generated and/or analyzed during the current study are available from the corresponding author on reasonable request.

## Abbreviations

CRE: cAMP 26 responsive element
CREB: CRE binding protein
DCX: Doublecortin
DTT: Dithiothreitol
ERK: Extracellular signal-regulated kinases
HIV-1: Human immunodeficiency virus-1
HSP: Heat shock protein
NSC: Neural stem cell
PBS: Phosphate-buffered saline
PCR: Polymerase chain reaction
pCREB: Phosphorylated CREB at Ser133
ROD: Relative optical density
T1: Sample trial
T2: Choice trial
